# Pediatric Transepiphyseal Seperation and Dislocation of the Femoral Head

**DOI:** 10.1155/2013/703850

**Published:** 2013-03-14

**Authors:** Mehmet Elmadag, Hasan H. Ceylan, Ahmet Can Erdem, Kerem Bilsel, Gokcer Uzer, Mehmet Ali Acar

**Affiliations:** ^1^Department of Orthopaedics and Traumatology, BezmialemVakif University Medical Faculty Hospital, 34093 Istanbul, Turkey; ^2^Department of Orthopaedics and Traumatology, Selçuk University Medical Faculty Hospital, 42075 Konya, Turkey

## Abstract

Pediatric hip fractures and dislocations are rare in practice and are related to high-energy trauma. The incidence of postoperative avascular necrosis is increasing, especially in the case of transepiphyseal fractures. Surgery is the most common form of treatment, and its timing is important for prognosis of the fracture. Patients and their families should be informed about the possibility of avascular necrosis and further complications related to the fracture.

## 1. Introduction

Pediatric hip fracture resulting from high-energy trauma is extremely rare in pediatric fractures and is an orthopedic emergency [1]. Transepiphyseal fracture of the femoral head accounts for only 8% of pediatric hip fractures [2]. Dislocation together with femoral head transepiphyseal fracture is an indication of high-energy trauma. Even when surgical treatment is successful, a high rate of avascular necrosis is seen in the femoral head. Nonetheless, premature closure of the epiphysis and coxa vara are frequent complications following these fractures [1, 3]. This situation requires particular explanation to patients and their families. A case is presented here of a patient treated for right-side traumatic hip dislocation with fracture, including femoral head posterior dislocation by separation from the epiphyseal line and acetabular posterior lip fracture and concomitant forearm fracture, resulting from high-energy trauma.

## 2. Case Presentation

A 10-year-old male patient presented at the emergency department 3 h after a traffic accident in which he was outside the vehicle. He was conscious and able to respond to verbal commands. He had extremely severe pain in the right hip and pain in the right forearm. On physical examination, there was shortness and external rotation in the right extremity.

**Figure 1:**
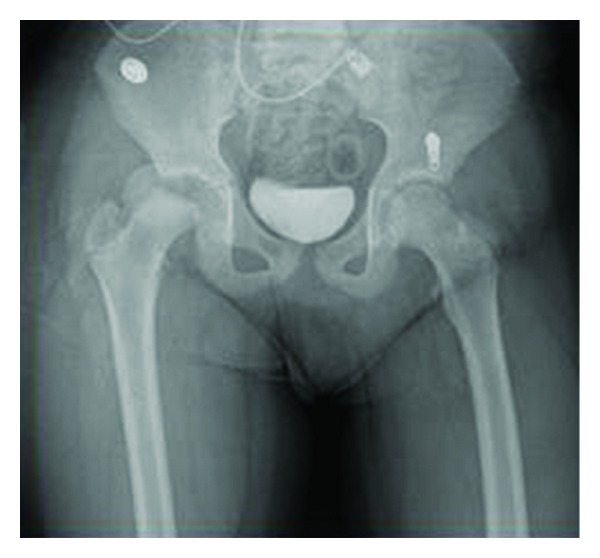
Patient admitted to our emergency service with right-sided proximal femoral fracture and dislocation.

**Figure 2:**
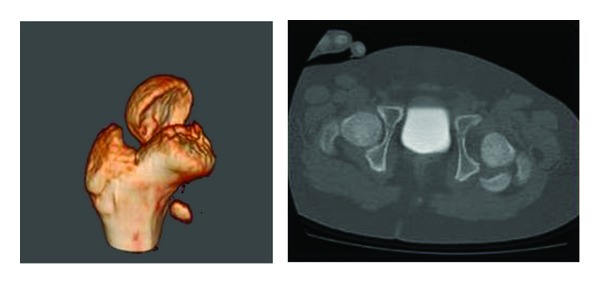
Preoperative computerized tomography imaging of the fracture.

Circulation in the extremity was completely normal. Radiological examination revealed a right forearm fracture and dislocation with transepiphyseal fracture of the right femoral head (Figure 1). On computed tomography, the femoral head was dislocated to the posterior of the acetabulum (Figure 2). The patient was admitted for surgery 2 h after presentation.

**Figure 3:**
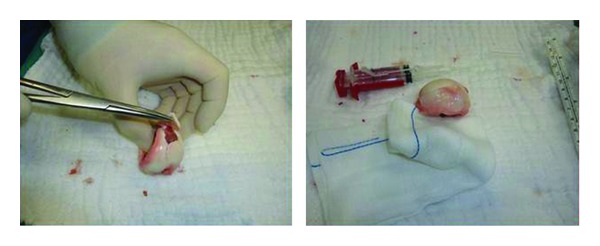
The chondral fragments sealed with fibrin glue before internal fixation.

The fracture line was reached with a posterolateral incision to the right hip. The femoral head was completely separated from the epiphyseal line and fracture fragments were displaced posteriorly. An area of 20% in the joint cartilage in the femoral head had damage in the form of a flap, so the damaged area was repaired with Tisseel sealant (Figure 3). By reduction of the femoral head, osteosynthesis was achieved with one 6.5 mm cannulated screw.

**Figure 4:**
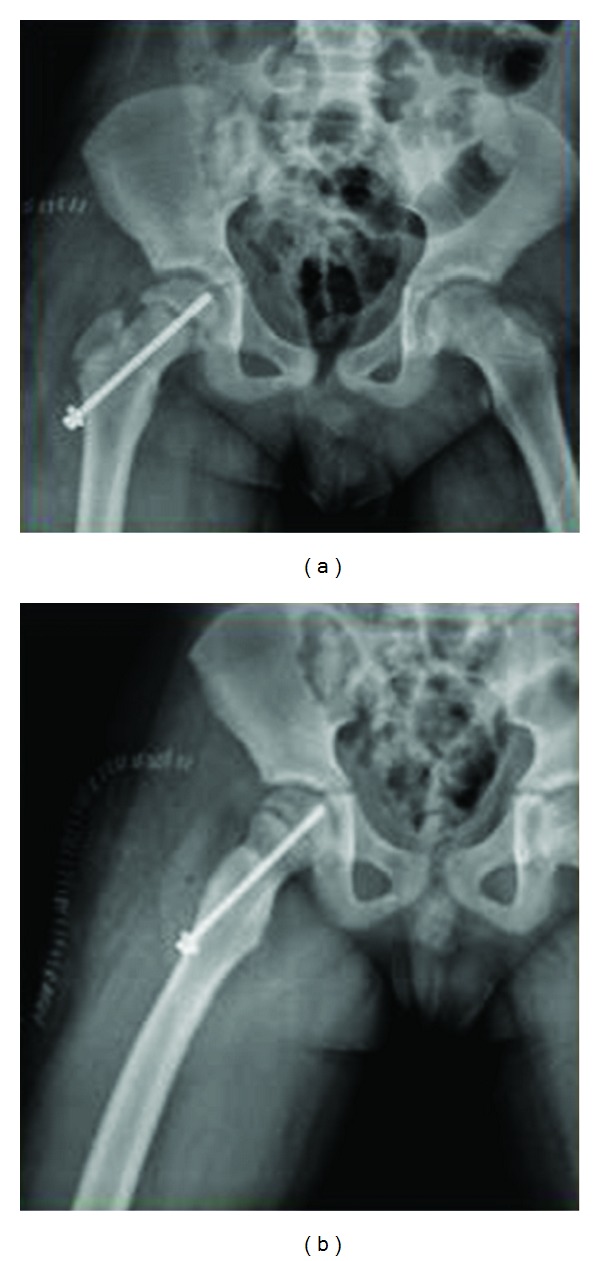
Postoperative (a) AP and (b) lateral views of related hip joint.

No fracture was seen on the acetabulum joint surface. The hip stability was checked and the acetabulum posterior lip was excised. Postoperatively, the hip joint was in position, and the relationship between the femoral head and the acetabulum was normal (Figures 4(a) and 4(b)). In the same session, open reduction and fixation with a K-wire were applied to the right forearm fracture.

On postoperative day 1, the patient was mobilized without weight bearing. Postoperatively, the motor sciatic nerve was seen to have been damaged. Traction would have been traumatic; therefore, using conservative treatment it was decided. A dynamic ankle splint was applied. Hip exercises were continued together with a physiotherapist, and there was non-weight-bearing mobilization for 6 weeks. At 3 months postoperatively, hip movement in external rotation was 10° limited and other movements were normal. At 6 months postoperatively, he was able to have full weight-bearing mobilization. The 10° limitation in external rotation continued. At the same time, the sciatic nerve damage and dropped foot had recovered completely.

**Figure 5:**
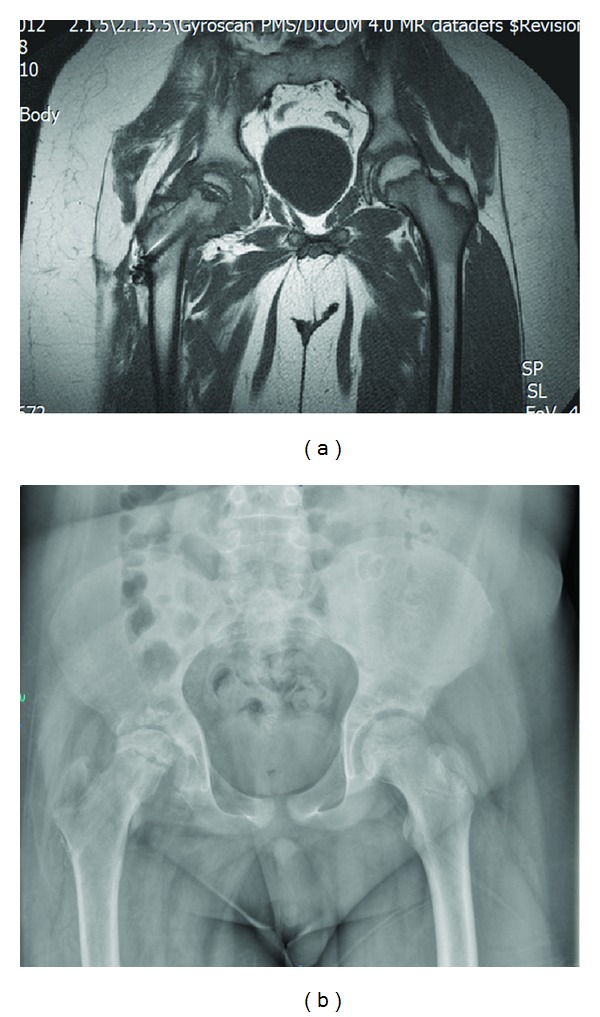
(a) One-year follow-up magnetic resonance imaging study shows no signs of avascular necrosis; (b) the screw removed after one year.

On magnetic resonance imaging at the 12-month follow-up, no avascular necrosis was seen to have developed in the femoral head (Figure 5(a)). After completion of fracture healing, the 6.5 mm cannulated screw was removed (Figure 5(b)). Under anesthesia, the joint movements were normal.

## 3. Discussion

Hip fractures include those in the area between the femoral head and the trochanter. Pediatric fractures of the femoral neck are extremely rare [2–5]. They generally occur as a result of high-energy trauma. These fractures peak particularly at two periods during childhood and from different causes: first, at a mean age of 7 years, from moderate-energy trauma of falling while participating in sports; and second, at ages 11–15 years, as a result of traffic accidents [6]. Factors that affect the prognosis include the type of trauma, type of fracture, and time to surgical intervention.

Avascular necrosis is known to be the most common complication that develops after these types of fractures [7–10]. This is because transepiphyseal fractures cause the greatest impairment of blood flow and the growth plate. In studies of traumatic transepiphyseal separation, the incidence of avascular necrosis has been reported to be 80%–100% [4, 5]. In the study by Moon, the most significant factors causing avascular necrosis were the type of fracture and patient age [1]. In cases that develop avascular necrosis, hip prosthesis and arthrodesis are significant treatment choices at a later age.

The timing of treatment is of great importance. Early reduction and fixation reduces the possibility of premature closure of the epiphyseal line and nonunion [2, 4, 11, 12]. Despite the severity of the trauma to the patient presented here, it is possible that avascular necrosis did not develop because of the early surgery at 5 h after trauma.

In the classification by Delbet, pediatric femoral neck fractures are anatomically placed under four headings [13]. However, this classification is insufficient, particularly for Type 1 fractures. Although avascular necrosis rarely develops in Type 1a fractures, it develops in almost all Type 1b fractures. This is because in Type 1b transepiphyseal separation, the femoral head may be dislocated in the acetabulum. In this situation, avascular necrosis that affects blood flow and the growth plate (epiphysis) may cause cessation of growth [8, 10]. The case presented here had a Delbet Type 1b femoral head transepiphyseal fracture. Also, the femoral head was dislocated to the posterior and there was a fracture of the acetabulum posterior lip.

In the literature, treatment choices have generally stayed with open reduction and internal fixation. However, some authors have presented closed reduction as an important treatment choice [5, 13]. In the present case, open reduction and internal fixation were applied with a posterior approach. The fractured area of the acetabulum lip was very small, and after determining that the safe zone range was good, fixation was not achieved and it was excised.

In a 1-year postoperative followup of the patient presented here, no radiological or clinical complications were encountered. We believe that timing and appropriate surgical treatment of these kinds of patients can overcome the development of avascular necrosis. However, the possibility of avascular necrosis developing in the femoral head and the potential prognosis should be explained in detail to patients and their families.
